# Latissimus Dorsi Flap in the Treatment of Thoracic Wall Defects After Medial Sternotomy

**Published:** 2020-05-29

**Authors:** Adam Stepniewski, Joelle Krahlisch, Alexander Emmert, Ahmad-Fawad Jebran, Maximilian Schilderoth, Helen Synn, Gunther Felmerer

**Affiliations:** ^a^Division of Plastic Surgery, Department of Trauma Surgery, Orthopedics and Plastic Surgery; ^b^Department of Thoracic and Cardiovascular Surgery, University Medical Center Goettingen, Goettingen, Germany

**Keywords:** median sternotomy, sternal infection, latissimus flap, surgical therapy, complications

## Abstract

**Background:** This study aimed to describe the subjective and objective results of the latissimus dorsi muscle flap and propose it as a reconstructive option for postoperative thoracic defects. **Methods:** A systematic search for cases with pedicle-based latissimus dorsi flaps performed after medial sternotomy was conducted, and all cases occurred between 2010 and August 2017. Preoperative, intraoperative, and postoperative factors were retrospectively analyzed and then the correlations between prognostic factors and outcomes of flap surgery were calculated. Furthermore, an evaluation of the subjective quality of life after flap surgery was performed using questionnaires. **Results:** A total of 25 cases were identified (8 female and 17 male patients) with the mean age of 75.28 years (range, 55-88 years). The average survival rate was 39.63 ± 23.03 months. The proportion of patients with a survival rate of 1 year was 84.00% (21 patients), and the proportion of patients with a 2-year survival rate was 80.00% (20 patients). While 24% of all patients who had latissimus dorsi flap operations experienced no complications, 64% of them developed minor complications (non–life-threatening, Clavien-Dindo grades I-IIIb) and 12% of them developed major complications (life-threatening, Clavien-Dindo grades IV-V). There was a significant correlation between the low survival rate and risk factors such as a positive history of smoking (*P* = .034), renal insufficiency (*P* = .022), metabolic syndrome (*P* = .004), and the presence of postoperative complications (*P* < .00002). No significant correlation was observed between the survival rate and obesity (*P* = .396), hyperlipoproteinemia (*P* = .684), arterial hypertonia (*P* = .0450), diabetes (*P* = .891), cardiovascular comorbidities (*P* = .794), the interval between sternotomy and latissimus flap surgery (*P* = .075), the duration of flap surgery (*P* = .207), sternal osteitis (*P* = .78), and intraoperative application of norepinephrine (*P* = .818). We identified metabolic syndrome (hazard ratio: 6.27), renal insufficiency (hazard ratio: 3.935), and the presence of postoperative complications (hazard ratio: 2.965) as high-risk prognostic factors. The subjective evaluations revealed positive reports from the patients with an average score of 1.86 ± 1.03 (1.0 = very good; 5.0 = poor). **Conclusions:** The majority of the patients with defects after median sternotomy were treated successfully with the latissimus dorsi flap. High survival rates, low rates of severe complications, and subjective scoring of improved life quality make this procedure relative safe and reliable. However, some prognostic risk factors limit the outcome, so these factors should be considered during surgical planning.

The advances in medical care, as well as the prolonged life expectancy, nowadays have led to an increased number of elderly and multimorbid patients who need cardiothoracic treatment. This leads to an increasing number of postoperative complications, such as wound-healing disorder and sternal osteomyelitis, which can result in high postoperative morbidity and mortality.^1^ The mortality rate of patients with postoperative sternal defects is up to 50%, if left untreated.^2^^,^^3^

Thoracic wall defects can cause a life-threatening problem, so the therapeutic concept should consider various mechanical and functional aspects. The defect has to be covered with healthy tissue to enhance the local perfusion, allowing the wound to heal. Simultaneously, the therapy needs to preserve vital mediastinal structures and to restore thorax stability for respiratory function.^3^

When designing their therapeutic plans, surgeons have to understand the anatomical and physiological aspects related to the defect of the thoracic cavity, and different surgical techniques can be applied to cover wounds in reconstructive surgical procedures.

This study aimed to describe the subjective and objective results of a single reconstructive procedure (latissimus dorsi muscle flap surgery) for thoracic defects, as this is one of the flap techniques commonly used for chest wall reconstruction. The preoperative, intraoperative, and postoperative factors affecting the outcome are identified along with their correlations to the survival rate. Furthermore, prognostic risk factors are evaluated.

## METHODS

A retrospective cohort study was conducted; the resulting data were extracted from the electronic, photographic, and paper-based documentation, including records stored between January 2010 and September 2017. Only patients treated with pedicled-based latissimus dorsi flaps due to postoperative sternal wounds after median sternotomy were included. Preoperative, intraoperative, and postoperative data were collected with standardized protocols and were analyzed accordingly. We considered the following parameters as risk factors: obesity, tobacco smoking, and the presence of renal insufficiency, diabetes, arterial hypertonia, hyperlipoproteinemia, cardiac comorbidities, metabolic syndrome, sternal osteitis, interval between sternotomy and latissimus flap surgery, duration of flap surgery, an application of intraoperative high-dose norepinephrine, and occurrence of postoperative minor or major complications. We calculated, whether the risk factors and complications classified after Clavien and Dindo^4^^,^^5^ have correlations to the survival rates. Furthermore, prognostic factors were identified. To assess the subjective outcome, we designed questionnaires for the patients, and the patients completed the survey in our outpatient clinic.

All reconstructive surgical procedures were performed by 2 surgeons from 2010 to 2017. All the data collected were statistically evaluated with univariate statistical tests (χ^2^ test, *t* test, and Kaplan-Maier analysis with the Mantel-Cox log-rank test) and multivariate logistic regression analysis (Cox regression). Statistical analyses were conducted using the IBM SPSS Statistics version 24.0.0.0 software. The data were anonymized according to the WMA Declaration of Helsinki, with the approval of the ethical commission (number 08/10/17). Informed consent from patients was obtained.

## RESULTS

**Figure 1:**
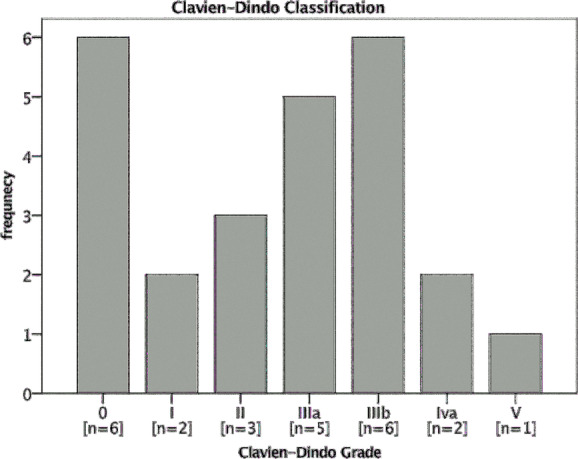
Bar graphic Clavien-Dindo classification.

**Figure 2:**
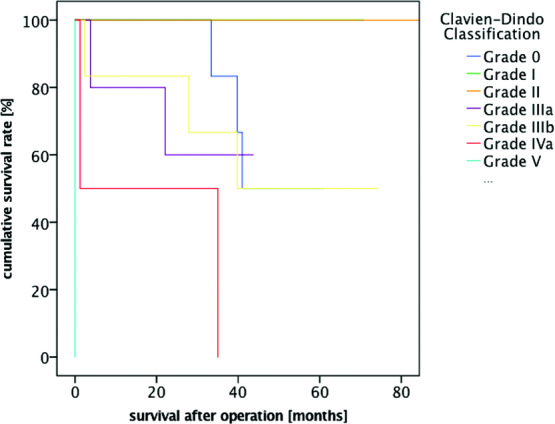
Kaplan-Meier survival curve postoperative complications.

A total of 25 patients who received latissimus dorsi flaps through reconstructive surgical procedures (8 female and 17 male patients, mean age: 75.28 years; range, 55-88 years) were included in our study. The proportion of patients with a survival rate of 1 year was 84.00% (21/25 patients), and the proportion of patients with a 2-year survival rate was 80.00% (20/25 patients). When the evaluation was completed, 11 of 25 patients had died. While 24% of the operated patients had no complications, 64% of them developed minor complications (non–life-threatening, Clavien-Dindo grades I-IIIb) and 12% of them developed major complications (life-threatening, Clavien-Dindo grades IV-V) (Tables 1 and 2; Fig 1). There was a strong correlation between low survival rates and the presence of postoperative complications (*P* < .00002) (Fig 2).

**Figure 3:**
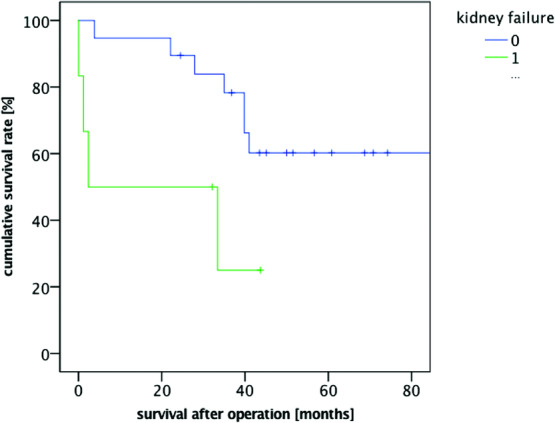
Kaplan-Meier survival curve renal insufficiency.

**Figure 4:**
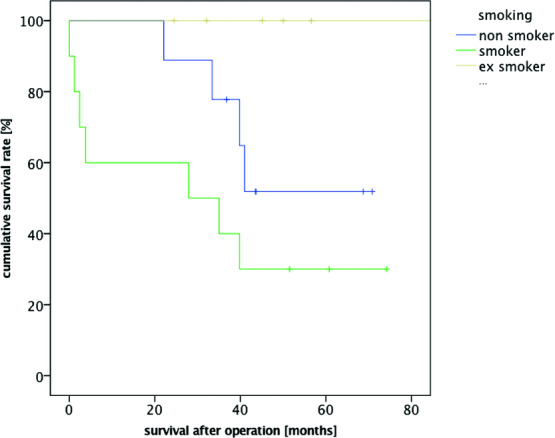
Kaplan-Meier survival curve smoking.

**Figure 5:**
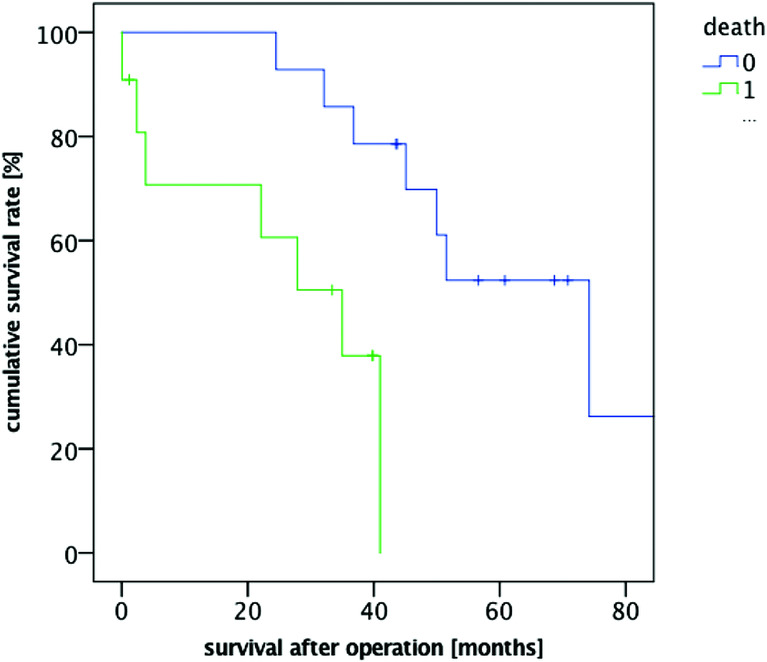
Kaplan-Meier survival curve effect of metabolic syndrome.

The following risk factors also showed a significant correlation for decreased postoperative survival rates: the presence of preexisting renal insufficiency (*P* = .022) and a positive history of smoking (*P* = .034) (Figs 3 and 4). Concerning the latter, it did not matter whether the patient was an active smoker at the time of flap surgery or he or she had only been a smoker in the past. However, we could not demonstrate a correlation between the history of smoking and the occurrence of flap tissue necrosis or wound-healing delays (*P* = .791). While the presence of metabolic syndrome had a significant correlation with decreased survival rates (*P* = .004) (Fig 5), it did not have a correlation with the occurrence of complications (*P* = .842).

No statistically significant correlation was found between the presence of obesity (grade I vs grade II vs grade III vs grade IV: 42.10 ± 30.07 months vs 37.22 ± 19.51 months vs 41.07 ± 21.83 months vs 38.30 ± 50.77 months, respectively; *P* = .396) and the survival rate. Patients with comorbidities lived for a shorter period than the patients without comorbidities (patients with diabetes: 36.65 ± 25.66 months vs patients without diabetes: 44.93 ± 17.51 months; patients with sternal osteitis: 38.70 ± 13.54 months vs patients without sternal osteitis: 45.59 ± 28.12 months), but the difference was not statistically significant (*P* = .891 and *P* = .78, respectively).

The average interval between sternotomy and latissimus flap surgery was 72.16 ± 72.73 days. Within this period, several debridements were performed (mean: 6.8 ± 5.92 debridements). Patients were sometimes later referred to the plastic surgery department and required to have flap surgery because of exposed ribs and instable breathing. Concerning this interval, no significant influence on the survival rates could be detected (*P* = .075). Patients who underwent flap surgery 72 to 152 days (group 2) after sternotomy lived longer than the patients who had the surgery before the 72nd day (group 1) and after the 152nd day of sternotomy (group 3) (group 1 vs group 2 vs group 3: 35.40 ± 24.10 months vs 61.33 ± 14.01 months vs 36.07 ± 3.33 months, respectively).

The average duration of flap surgery was 248 ± 86 minutes. Patients were categorized into 3 groups, based on surgery duration: 160 to 220 minutes (group 1; 12 patients), 221 to 280 minutes (group 2; 5 patients), and longer than 280 minutes (group 3; 8 patients). There was no significant difference concerning survival rates among these 3 groups. However, individuals in group 2 had the highest survival rate (group 1 vs group 2 vs group 3: 37.96 ± 6.82 months vs 77.00 ± 8.05 months vs 48.64 ± 9.26 months; *P* = .207).

The intraoperative application of norepinephrine did not have a significant influence on postoperative survival rates (*P* = .818). The subjective evaluation showed better results in early follow-up inpatient treatment after surgery (1.86 ± 1.03) than the late follow-up care in September 2017 (1.93 ± 1.21; 1.0 = very good, 5.0 = poor). We evaluated patient satisfaction and arrived at subjective scorings based on changes in the quality of the patients’ lives and of their respiratory function. Three patients (12.00%) reported subjective postoperative alterations in breathing, and 2 of these 3 patients (66.67%) suffered from dyspnea. While taking deep breathing, one of these patients suffered pain due to discomfort in the medial ends of the clavicles. Consequently, he avoided taking deep inhalations, which further led to decreased respiration rate and physical resilience.

## DISCUSSION

The most common method for closure of defects after medial sternotomy is the use of pedicled latissimus dorsi flap. The architecture of this myocutaneous flap^6^^,^^7^ allows for the reconstruction of the defect with the possibility of filling large cavities with well-perfused muscle, thus protecting the area from bacterial contamination and accelerating tissue recovery.^8^

In our study, the proportion of patients with a survival rate of 1 year was 84.00% (21/25 patients) and the proportion with a 2-year survival rate was 80.00% (20/25 patients), and these results correspond with those from other literature.^9^

Moreover, we demonstrated a significant association between postoperative complications and decreased survival rates. Both early and late complications had a strong influence on the survivability (*P* < .00002). We calculated the presence of postoperative complications with a hazard ratio of 2.965 (95% confidence interval 0.845–3.13), making it one of 3 high-risk prognostic factors.

The most influential factor of the survival rate was the presence of acute renal failure,^10^ either as an isolated condition or due to preexisting renal insufficiency (*P* = .00003); lethality for patients who underwent treatment was up to 70%.

According to Krug et al,^11^ complication rates are increased when the person smokes more than 20 cigarettes a day, but occasional smokers or ex-smokers do not have an increased risk of wound-healing delays or local necroses. Based on our results, a significant correlation between nicotine consumption and lower survival rate (*P* = .034) was observed but not between necroses or wound-healing delays and lower survival rates (*P* = 0.791). However, a small number of patients could have biased our results (10 active smokers, mean = 30.00 ± 8.66 pack-years; 6 ex-smokers, mean = 38.33 ± 24.01 pack-years).

The fourth factor, which showed a significant correlation for diminished survival rates, was the metabolic syndrome (*P* = .004). In addition, the mean survival rate of patients with the metabolic syndrome was 35.50 ± 24.44 months whereas that of patients without the metabolic syndrome was 45.83 ± 20.31 months in our study. Metabolic syndrome can lead to complications because the weight of fat tissue and skin can cause longitudinal or perpendicular traction on the wound edges and prevent tissue perfusion. Furthermore, it may interfere with surgical suturing.^12^ Although a significant correlation between metabolic syndrome and the occurrence of complications was not found in our study (*P* = .842), we propose that the presence of the metabolic syndrome can be treated as a negative prognostic factor (hazard ratio: 6.27; 95% confidence interval, 1.53–25.62).

The grade of local infection or the presence of sternal osteitis depends on the extent of the debridement surgery. The insufficient debridement and infection control can lead to enlargement of the sternal defect and create the unstable thorax wall. The surgical time can be consequently much longer if the defect requires a larger latissimus flap.^13^ According to our results, insufficient debridement and the presence of persistent osteitis decreased the survival rate (*P* = .780). The patients who had minor infection had the highest survival rate (45.59 ± 28.12 months). Interestingly, patients with pronounced local infection lived longer than those with moderate infection (38.70 ± 13.54 months vs 36.78 ± 23.96 months). Thus, we can hypothesize that large infections can also be treated successfully. However, it requires sufficient debridement.

Further investigation of a potential correlation between the length of time between sternotomy and flap surgery and the survival rate is needed (*P* = .075). Patients with the sternal defect who were operated on to create the latissimus flap 76 to 152 days after sternotomy had the greatest survival rate of 61.33 ± 14.01 months. A lower patient survival rate was found when the period between 2 operations was longer than 152 days (36.07 ± 3.33 months). Unfortunately, it is not clear whether the vacuum-assisted therapy was employed as an additional process to prepare the target area for latissimus flap surgery or as an exclusive method prior to flap surgery. In the other studies, it is stated that a vacuum-assisted closure therapy can improve the overall outcome of treatment.^14^

In our study, the period during which the complete surgery occurred was remarkably long. Therefore, it is important to investigate whether an abbreviated period between surgical procedures could have improved the outcome. The patients were transferred from another department, and flap surgery was completed immediately after the takeover. The flap operation was carried out by plastic surgeons on average a week after the takeover from another department.

An important feature of our study is that the questionnaires were formulated for patients to evaluate their respiratory function and quality of life. Most of the patients did not report changes in their breathing patterns after sternotomy or a combination of sternectomy and flap surgery. However, it was only a subjective assessment and no objective evaluation, such as pulmonary function testing, was done. This can be one of the limitations of our study.

In the literature, there were no differences in pulmonary function between the cases with conventional sternotomy and those with complete sternectomy without stabilizing the thoracic wall.^15^ Leaving a wide bony gap after sternectomy can prevent painful friction, which can reduce the patient's quality of life.^16^ Because one of our patients reported this chest pain after the sternectomy, it was important to consider this procedure in our study. Furthermore, 2 patients reported dyspnea, especially during physical activity, which was described as impairing the quality of life.

The overall subjective evaluation showed more positive results in early follow-up inpatient treatment after surgery (1.86 ± 1.03) compared with the later follow-up care in September 2017 (1.93 ± 1.21; 1.0 = very good, 5.0 = poor). One of the other factors, which could have contributed to a positive subjective outcome, was the relatively short hospital stay after flap surgery. Compared with those who experienced a longer stay due to complications resulting from sternotomy, the patients who had relatively quick recovery after latissimus flap surgery reported it as a great benefit to them.

## CONCLUSIONS

Based on our experience, we can suggest the efficacy of pedicle-based latissimus dorsi flaps in the treatment of sternal defects. The majority of the patients in our study were treated successfully with this technique. High survival rates, low rates of severe postoperative complications, and individually reported scorings of life quality suggest this procedure to be safe and reliable. However, some prognostic risk factors limit the outcome, which should be considered in surgical planning.

We are aware of some limitations of our study. Only a relatively small number of cases were included. This can potentially create a bias against other therapeutic strategies, especially against other flap techniques. Further research, including ones with more cases, is still needed.

## Figures and Tables

**Table 1:** Clavien-Dindo classification*

Grade I	Every deviation from the normal postoperative course without the need for pharmacological treatment or surgical, endoscopic, and radiological interventions. Accepted therapeutic regimens are drugs (analgetics, antiemetics, antipyretics, diuretics, and electrolytes), physiotherapy, and bedside debridement.
Grade II	Requiring pharmacological treatment with drugs other than the ones included in grade I. In this grade, blood transfusion and parenteral nutrition are also included.
Grade III	Necessity of surgical, endoscopic, or radiological intervention:
a	- Under locoregional anesthesia
b	- Under general anesthesia
Grade IV	Life-threatening complications, including central nervous system, requiring therapy within the ICU setting:
a	- One-organ failure
b	- Multiple-organ failure
Grade V	Death of the patient
“d”	The suffix d (“d” for disability) is used in patients who are discharged with a complication. Follow-up is required to comprehensively evaluate the outcome and related long-term quality of life. Close follow-up is necessary.

*ICU, indicates intensive care unit.

**Table 2:** Detailed overview of complications

Grade	Complications	Frequency, n	Sex		Percentage
			M	F	
0	None	6	5	1	24
I	Hematoma	2	1	1	8
II	Superficial necrosis of the skin	2	2	0	8
	Small seroma	1	0	1	4
IIIa	Seroma	5	2	3	20
IIIb	Fistula	2	0	2	8
	Necrosis of the skin	3	2	1	12
	Wound-healing disorder	1	1	0	4
IVa	Respiratory failure	2	2	0	8
IVb	…	…	…	…	…
V	Exitus letalis	1	1	0	4
